# Efficient formation of inert Bi-213 chelates by tetraphosphorus acid analogues of DOTA: towards improved alpha-therapeutics

**DOI:** 10.1186/s13550-018-0431-3

**Published:** 2018-08-08

**Authors:** Jakub Šimeček, Petr Hermann, Christof Seidl, Frank Bruchertseifer, Alfred Morgenstern, Hans-Jürgen Wester, Johannes Notni

**Affiliations:** 1 Lehrstuhl für Pharmazeutische Radiochemie, Technische Universität München,, Walther-Meißner-Strasse 3, 85748 Garching, Germany; 2 Department of Inorganic Chemistry, Charles University, Hlavova 2030, 12843 Prague 2, Czech Republic; 3 Department of Nuclear Medicine and Department of Obstetrics and Gynecology, Technische Universität München, Munich, Germany; 4 European Commission, Joint Research Centre, Directorate for Nuclear Safety and Security, Karlsruhe, Germany; 5 Present address: Isotope Technologies Garching GmbH, Garching, Germany

**Keywords:** Bismuth, Phosphonic acid, Phosphinic acid, Radiopharmaceuticals, Targeted alpha therapy

## Abstract

**Background:**

The recently growing interest in targeted alpha-therapy (TAT) calls for improvement of the labelling chemistry of the corresponding radionuclides. ^213^Bi^III^ is a short-lived alpha emitter which emits only one alpha particle in its decay chain. Hence, it might be safer in application than other respective nuclides, such as ^223^Ra or ^225^Ac, because no alpha-emitting daughters are released upon recoil. We investigated cyclen derivatives with phosphorus-containing pendant arms regarding their suitability for ^213^Bi labelling.

**Results:**

The concentration dependency of ^213^Bi labelling at 25 °C and 95 °C was determined for DOTP, DOTP^H^, DOTP^Et^, and DOTPI, as well as for DOTA and CHX-A"-DTPA for comparison. The labelling efficiency of the phosphorus-containing ligands was at least comparable to CHX-A"-DTPA and exceeded that of DOTA. DOTP was most efficient, requiring chelator concentrations for labelling which were approx. two orders of magnitude lower than those required for CHX-A"-DTPA, both at 25 °C and 95 °C. The ^213^Bi complexes of phosphorus ligands furthermore showed a higher stability against demetallation (> 96% of intact complex after 120-min incubation in plasma were found for DOTP, DOTP^H^, and DOTP^Et^, compared to 85% for DOTA and 76% for CHX-A"-DTPA).

**Conclusion:**

Cyclen derivatives bearing four *N*-methylenephosphonic or -phosphinic acid substituents, e.g., DOTP, are capable of complexing the alpha-emitting radionuclide ^213^Bi^III^ with higher efficiency and in-vitro stability than the current gold standards DOTA and CHX-A"-DTPA.

## Background

Compared to β- or γ-radiation, tissue interaction of α-particles is characterized by a higher linear energy transfer (LET) and a much higher cell toxicity due to an enhanced probability of causing DNA double strand breaks [1]. In addition, the low tissue penetration depth of α-radiation (3–4 cell diameters) entails a more localized therapeutic effect, ideal for killing remaining single cancer cells or micrometastases which, in most conventional treatment regimes, can survive and later function as nuclei of tumor recurrence. Nevertheless, radionuclide therapy of cancer using radiopharmaceuticals labeled with α-emitting radionuclides (referred to as “targeted alpha therapy,” TAT) has hitherto played only a limited clinical role, although the therapeutic potential of the α-emitter ^225^Ac (*T*_½_ = 9.92 *d*) [2] has been emphasized already in 2001 [3]. The recent approval and market entry of ^223^Ra chloride as an α-emitting therapeutic radiopharmaceutical [4] and successful application of ^225^Ac-labeled inhibitors of prostate-specific membrane antigen (PSMA) for treatment of prostate cancer [5] highlighted the clinical potential of α-therapy and led to a tremendous boost of attention for TAT.

However, despite of proven suitability of ^225^Ac for treatment of terminal cases, such as β-refractory prostate cancer patients [5], there are still concerns regarding safe applicability of this nuclide for other than palliative use. There are four α-decays in its multistep decay scheme, while the recoil of the first α-emission releases the nuclide from the binding site (typically a chelate) [6]. In adverse cases, slow or incomplete internalization into cells may lead to uncontrolled distribution of the various α-emitting daughter nuclides in the body, causing the risk of severe side effects (e.g., kidney toxicity or carcinogenesis) owing to undesirable irradiation of healthy tissue [7]. In view of these issues, ^213^Bi (*T*_½_ = 46 min) [8], a late daughter nuclide of ^225^Ac, appears to be a valuable alternative. Diffusion after recoil is not a problem because a stable isotope, ^209^Bi, is obtained after decay via nearly simultaneous α- and β-emissions, while an additional 440 keV γ-line enables scintigraphic imaging. ^213^Bi is conveniently obtained from ^225^Ac/^213^Bi generators, small shielded chromatographic benchtop devices containing ^225^Ac^III^ adsorbed to an organic matrix, from which ^213^Bi^III^ is eluted with iodide solution in form of the [^213^BiI_4_]^−^ and [^213^BiI_5_]^2−^ complexes [9]. Hence, ^213^Bi has been exploited for various therapeutic applications [10–12], none of which, however, reached clinical routine so far, above all, due to very limited availability of ^213^Bi. Nevertheless, the awakened interest in ^225^Ac and the foreseeable expansion of global ^225^Ac production capacity will also entail a wide availability of ^213^Bi generators in the near future [13]. Overall, a higher inherent safety of ^213^Bi, resulting from its short half-life and a decay scheme involving only a single α-decay, renders this nuclide attractive for future development of α-therapeuticals which, in turn, calls for improvement of the corresponding labelling chemistry that hitherto received only little attention.

## Materials and methods

### General

*p*-NH_2_-CHX-A"-DTPA was purchased from Macrocyclics (Plano, TX, USA). 1,4,7,10-tetraazacyclododecane-1,4,7,10-tetraacetic acid (DOTA) was purchased from CheMatech (Dijon, France). Previously published, optimized protocols were applied for synthesis of the chelators 1,4,7,10-tetraazacyclododecane-1,4,7,10-tetrakis[methylene(2-carboxyethylphosphinic acid)] (DOTPI) [14] as well as for 1,4,7,10-tetraazacyclododecane-1,4,7,10-tetrakis(methylenephosphonic acid) (DOTP), 1,4,7,10-tetraazacyclododecane-1,4,7,10-tetrakis(methylenephosphinic acid) (DOTP^H^), and 1,4,7,10-tetraazacyclododecane-1,4,7,10-tetrakis[methylene(phosphonic acid monoethyl ester)] (DOTP^OEt^) [15].

Radio-TLC was performed on glass microfiber chromatography papers impregnated with silica (ITLC®, Agilent). Using 0.1 M aq. sodium citrate as eluent, non-incorporated ^213^Bi^III^ is transformed into the citrate complex which moves with the solvent front, while all chelates are sufficiently retained to enable ground-line separation (*R*_f_ < 0.5). Readout of chromatograms was done using a Bioscan TLC scanner, consisting of B-MS-1000 scanner, and B-EC-1000 detector with a B-FC-3600 GM tube.

### ^213^Bi labelling

^213^Bi^III^ was eluted with a mixture of 0.2 M aq. HCl (0.3 mL) and 0.2 M aq. sodium iodide (0.3 mL) as anionic species [^213^BiI_4_]^−^ and [^213^BiI_5_]^2−^) from a^225^Ac/^213^Bi generator system with an initial activity of 150 MBq as provided by the Institute for Transuranium Elements (Karlsruhe, Germany) [16]. The eluate was adjusted to pH 5.5 with 1 M aq. NaOAc buffer (1.6 mL). Labelling was performed by addition of the buffered eluate (90 μL) into an Eppendorf cup containing the precursor solution (10 nM–1 mM, 10 μL), resulting in final chelator concentrations of 0.001–100 μM. After 5 min of incubation at ambient temperature (approx. 25 °C) or at 95 °C, the fraction of complexed ^213^Bi^III^ was evaluated by radio-TLC.

### Stability studies

Stability of ^213^Bi^III^-complexes was tested in human plasma or 0.1 M aq. Na-DTPA (pH 7.5) by addition of 10 μL of the labelling solution (with 1 mM ligand concentration), containing the radiometal complex, to 90 μL of the competing medium at 37 °C. The fraction of intact chelate was evaluated by radio-TLC. Values were normalized to the fraction of radiometal complex at *t* = 0.

## Results

**Fig. 1:**
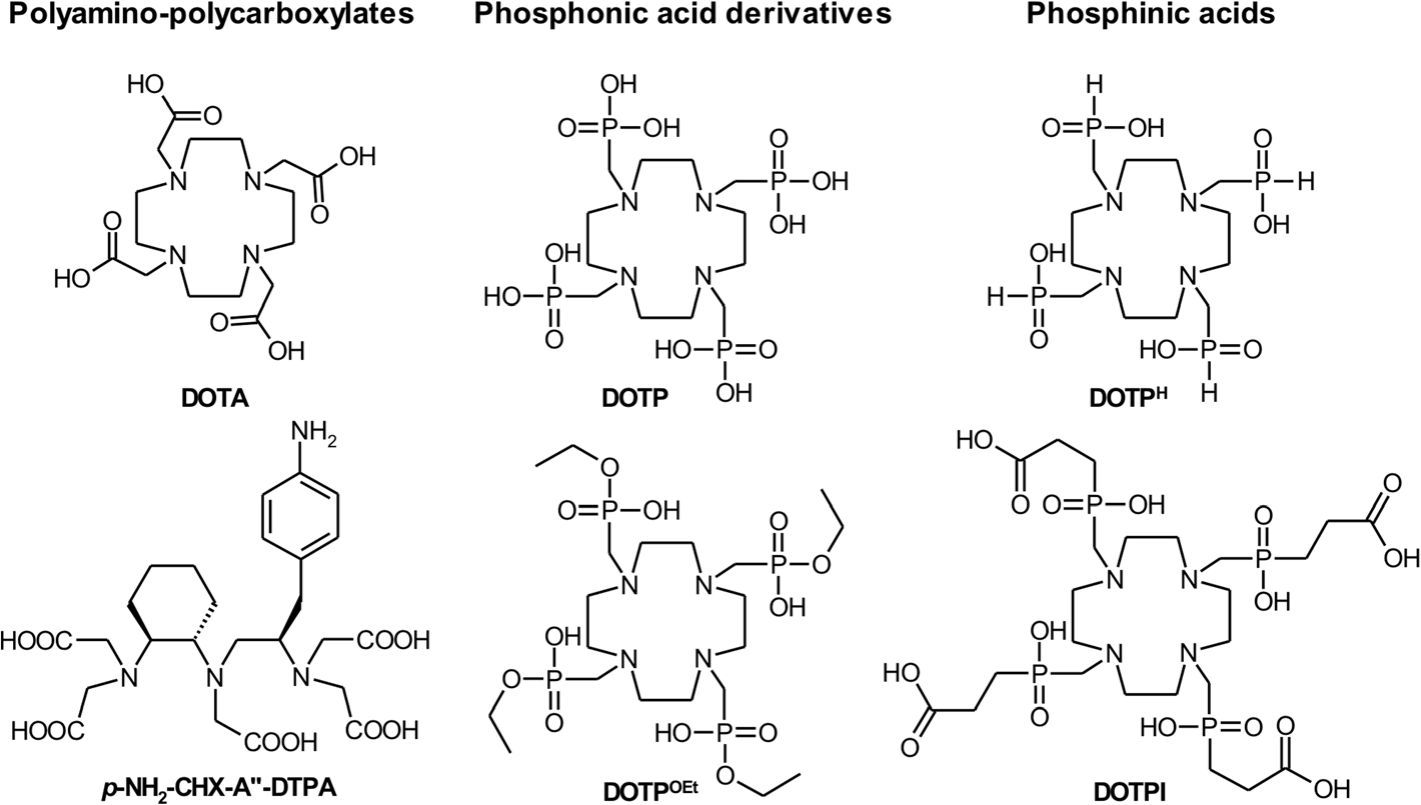
Chelator motifs investigated in terms of ^213^Bi^III^ complexation properties

Up to now, ^213^Bi^III^ labelling virtually exclusively relied on well-established acyclic or cyclic polyamino-polycarboxylate ligands, above all, CHX-A"-DTPA [17] or the highly popular and versatile chelator DOTA [18] (Fig. 1). However, we previously noticed that 1,4,7-triazacyclononanes bearing phosphinic acids as *N*-substituents (TRAP chelators) [19] show superior labelling efficiency for the short-lived trivalent positron emitter ^68^Ga^III^ [20, 21] in comparison to their parent tricarboxylate NOTA (1,4,7-triazacyclononane-1,4,7-triacetic acid) [22], pointing at potentially superior radiolabelling properties of phosphorus-pendant azamacrocycles in general. Thus, we elucidated the potential of cyclen-based chelators with phosphorus-based *N*-pendant arm donors for ^213^Bi^III^ complexation.

**Fig. 2:**
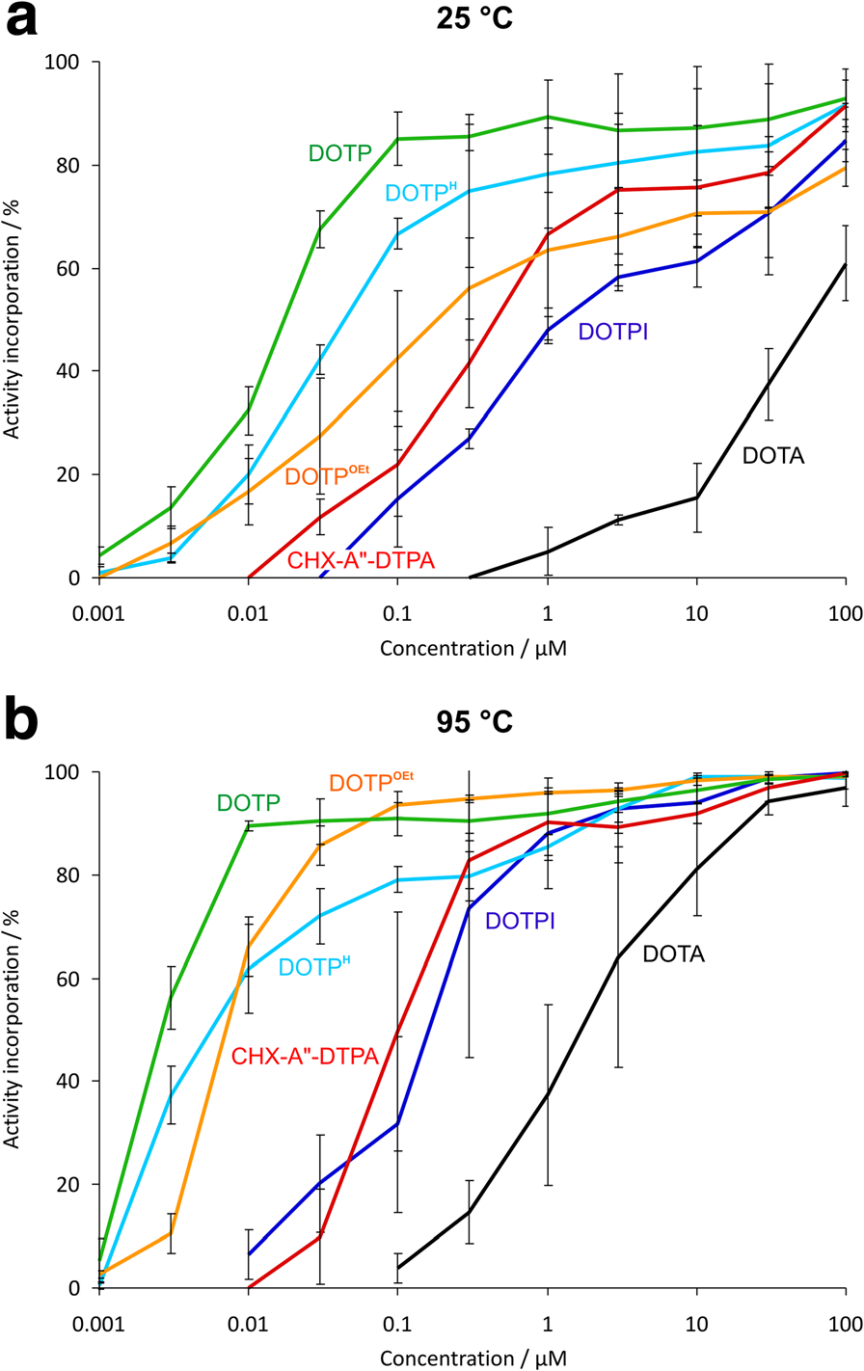
Incorporation of ^213^Bi^III^ by chelate ligands shown in Fig. 1 as functions of ligand concentration. Mean values ± SD, *n* = 3. Labelling conditions pH 5.5, *V* = 0.1 mL, reaction time 5 min, at 25 °C (**a**) and 95 °C (**b**). For data in numerical form, see Tables 1 and 2

For this purpose, ^213^Bi^III^ labelling of phosphonic acid derivatives DOTP [23] and DOTP^OEt^ [24] as well as of phosphinic acids DOTP^H^ [25] and DOTPI [14] was compared to the aforementioned standard scaffolds DOTA [26] and CHX-A"-DTPA [27], also under mild conditions (ambient temperature, pH 5.5) compatible with any type of biological targeting vector, including antibodies (Fig. 2; Tables 1 and 2). DOTA shows the poorest performance among all chelators investigated and, as with virtually all other radiometals, apparently cannot be labeled quantitatively with ^213^Bi within reasonable ranges of concentration and time at ambient temperature. This is why open-chain chelators, particularly CHX-DTPA derivatives, are usually applied for this purpose, despite of inherently lower in vivo stability of their M^III^ complexes [28]. However, to our surprise, the performance of phosphorus-based cyclens was found at least comparable to CHX-A"-DTPA, while DOTP showed particularly efficient radiolabelling, most likely due to higher affinity of trivalent bismuth to the relatively hard phosphonate oxygen atoms. This is a remarkable finding because in contrast to open-chain ligands, metal ion complexation by cyclic chelators usually occurs slower, via a two-step mechanism [29]. An initially formed *out-of-cage* complex, wherein the metal ion is coordinating only to side arm oxygen donors and solvent (water) molecules [25] is transformed into the *in-cage* complex, characterized by a N_4_O_4_ coordination mode of the ligand, via a substantial energy barrier. In terms of radiolabelling, this barrier causes a slower activity incorporation, but, on the other hand, is also related to higher kinetic inertness of the radiometal complexes which translates to lower dissociation rates.

**Table 1:** Percentage of incorporation of ^213^Bi^III^ by chelate ligands (pH 5.5, *V* = 0.1 mL, reaction time 5 min at 25 °C). Data represent mean values ± SD, *n* = 3

*c* [μM]	DOTP	DOTP^H^	DOTP^OEt^	DOTPI	CHX-A"-DTPA	DOTA
100	92.9 ± 5.6	91.6 ± 0.4	79.6 ± 3.6	84.8 ± 4.0	91.4 ± 5.1	61.0 ± 7.3
30	88.8 ± 10.7	83.8 ± 11.9	70.8 ± 8.8	70.6 ± 11.9	78.6 ± 6.9	37.4 ± 6.9
10	87.2 ± 11.8	82.5 ± 12.4	70.6 ± 6.5	61.5 ± 5.1	75.7 ± 11.8	15.6 ± 6.7
3	86.5 ± 11.2	80.4 ± 9.7	66.0 ± 9.5	58.1 ± 2.4	75.3 ± 12.6	11.2 ± 0.9
1	89.3 ± 7.2	78.4 ± 10.6	63.5 ± 11.2	47.9 ± 2.6	66.6 ± 20.6	5.1 ± 4.6
0.3	85.4 ± 2.5	75.0 ± 14.8	56.0 ± 9.9	27.0 ± 2.0	41.5 ± 8.5	
0.1	85.0 ± 5.2	66.7 ± 3.1	42.4 ± 13.2	15.4 ± 9.5	22.1 ± 10.1	
0.03	67.6 ± 3.5	42.2 ± 2.8	27.5 ± 11.2		11.8 ± 3.5	
0.01	32.4 ± 4.6	20.2 ± 5.7	16.7 ± 6.4			
0.003	13.6 ± 4.1	3.8 ± 0.9	6.6 ± 3.5			
0.001	4.3 ± 1.6	0.9 ± 1.3

**Table 2:** Percentage of incorporation of ^213^Bi^III^ by chelate ligands (pH 5.5, *V* = 0.1 mL, reaction time 5 min at 95 °C). Data represent mean values ± SD, *n* = 3

*c* [μM]	DOTP	DOTP^H^	DOTP^OEt^	DOTPI	CHX-A"-DTPA	DOTA
100	99.4 ± 0.3	98.7 ± 2.1	99.1 ± 0.1	99.7 ± 0.3	99.8 ± 0.1	96.9 ± 3.6
30	98.6 ± 0.9	99.0 ± 0.2	99.0 ± 1.0	98.8 ± 0.3	96.8 ± 2.4	94.3 ± 2.6
10	96.4 ± 2.3	99.1 ± 0.7	98.3 ± 0.9	94.2 ± 4.2	91.9 ± 1.9	81.1 ± 9.0
3	94.3 ± 2.1	92.7 ± 2.5	96.4 ± 0.6	92.9 ± 4.8	89.2 ± 6.9	64.1 ± 21.4
1	91.8 ± 4.0	85.4 ± 2.6	95.9 ± 0.3	88.0 ± 10.8	90.2 ± 6.6	37.4 ± 17.5
0.3	90.6 ± 3.9	79.7 ± 4.7	94.7 ± 0.7	73.6 ± 29.0	82.7 ± 5.3	14.6 ± 6.1
0.1	90.9 ± 3.2	79.0 ± 2.5	93.7 ± 2.6	31.7 ± 17.1	49.7 ± 23.1	3.8 ± 2.8
0.03	90.4 ± 4.5	72.0 ± 5.4	85.7 ± 3.9	20.2 ± 9.4	9.8 ± 9.2	
0.01	89.5 ± 1.0	61.8 ± 8.7	66.2 ± 5.8	6.5 ± 4.7		
0.003	56.2 ± 6.1	37.3 ± 5.5	10.5 ± 3.7			
0.001	5.3 ± 4.2	0.4 ± 0.6	2.5 ± 0.7			

**Fig. 3:**
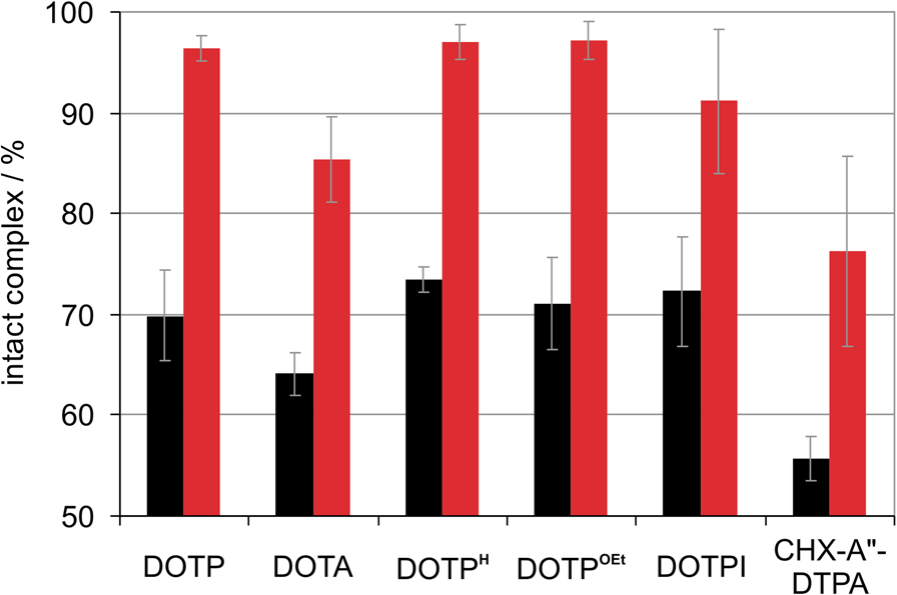
Stability of ^213^Bi chelates. Percentage of intact ^213^Bi^III^ complexes after 120 min challenge with 0.1 M aq. disodium DTPA at pH 7.5 (black bars) and in human plasma (red bars), both at 37 °C. For more data see Table 3

To assess this important parameter, we characterized the stability of the ^213^Bi chelates in a transchelation challenge against DTPA, and in human plasma at 37 °C. Figure 3 and Table 3 show that in accordance with expectations, the ^213^Bi^III^-complex of the open-chain ligand CHX-A"-DTPA exhibits the lowest kinetic inertness, resulting in a larger extent of dissociation than observed for the cyclic systems. Among the latter, all phosphorus ligands show quite similar resistance against demetallation. Notably, their ^213^Bi^III^ complexes are also more inert than that of DOTA, most likely because they are protonated at lower a pH [15, 30].

**Table 3:** Percentage of intact ^213^Bi^III^ chelates after incubation at 37 °C with 0.1 M aq. sodium DTPA (pH 7.5) or human plasma. Data represent mean values ± SD, *n* = 3

medium	*t* [min]	DOTP	DOTP^H^	DOTP^OEt^	DOTPI	CHX-A"-DTPA	DOTA
DTPA	30	97.3 ± 0.9	98.1 ± 0.8	94.6 ± 1.3	93.5 ± 0.6	92.5 ± 1.1	91.1 ± 2.4
DTPA	60	87.0 ± 7.9	87.1 ± 7.7	86.8 ± 6.3	83.3 ± 3.9	82.7 ± 8.1	83.4 ± 9.0
DTPA	120	69.9 ± 4.5	73.4 ± 1.2	71.0 ± 4.6	72.3 ± 5.4	55.6 ± 2.2	64.1 ± 2.1
Plasma	60	98.4 ± 1.6	99.2 ± 0.5	98.8 ± 0.8	95.6 ± 1.9	90.8 ± 4.6	96.1 ± 4.0
Plasma	120	96.4 ± 1.3	97.0 ± 1.8	97.2 ± 1.9	91.1 ± 7.1	76.3 ± 9.4	85.4 ± 4.3

## Discussion

With a > 90% stability in plasma over the entire dosimetrically relevant time period of ^213^Bi (approx. three half-lives), the phosphinate and phosphonate chelators appear better suited for a safe application in ^213^Bi therapeutics than CHX-A"-DTPA derivatives. In addition, the higher labelling efficiencies, i.e., lower molar amounts of chelator required for the same extent of radiometal incorporation, will provide radiopharmaceuticals with higher specific activity, that is, an improved ratio of labelled vs. non-labelled compound in the final preparation. Hence, by administration of the same amount of, e.g., a ^213^Bi labelled antibody, a multiple amount of activity could be deposited in the target (tumor) tissue, resulting in a substantially increased radiation dose per tissue volume and, consequently, in a more successful therapy.

## Conclusion

In conclusion, we found that ^213^Bi^III^ complexation properties of cyclen-based phosphinate and particularly of phosphonate ligands are superior to the gold standard acyclic or cyclic chelators for ^213^Bi^III^, CHX-A"-DTPA and DOTA, respectively, reaching comparable labelling yields at 2–4 orders of magnitude lower concentrations both at ambient and elevated temperatures. In view of such highly efficient ^213^Bi incorporation, the phosphorus chelators appear ideal for application in freeze-dried labelling kits as known from ^99m^Tc tracers and in antibody conjugates for immunotherapy where they would offer the benefits of improved in-vivo stability and higher target doses due to higher specific activity. Because at last, targeted α-therapy is widely entering clinical healthcare schemes after remaining in an experimental state for decades [13], our results are expected to support the currently increasing efforts towards advanced ^213^Bi radiotherapeutics for improved treatment of cancer.
